# Sensitivities of seven algal species to triclosan, fluoxetine and their mixtures

**DOI:** 10.1038/s41598-018-33785-1

**Published:** 2018-10-18

**Authors:** Ran Bi, Xiangfeng Zeng, Lei Mu, Liping Hou, Wenhua Liu, Ping Li, Hongxing Chen, Dan Li, Agnes Bouchez, Jiaxi Tang, Lingtian Xie

**Affiliations:** 1 Marine Biology Institute, Shantou University, Shantou, Guangdong 515063 China; 2 Key Laboratory of Pollution Ecology and Environmental Engineering, Institute of Applied Ecology, Chinese Academy of Sciences, Shenyang, Liaoning 110016 China; 3 School of Life Sciences, Guangzhou University, Guangzhou, Guangdong 510655 China; 4 The Environmental Research Institute, MOE Key Laboratory of Environmental Theoretical Chemistry, South China Normal University, Guangzhou, 510006 China; 5 Institute National de la Recherche Agronomique, UMR CARRTEL, Thonon-les-Bains, 74200 France; 6 College of Environmental Science and Engineering, Liaoning Technical University, Fuxin, Liaoning 123000 China

## Abstract

Increasing release of pharmaceuticals and personal care products (PPCPs) into aquatic ecosystems is a growing environmental concern. Triclosan and fluoxetine are two widely used PPCPs and frequently detected in aquatic ecosystems. In this study, the sensitivities of 7 algal species from 4 genera to triclosan, fluoxetine and their mixture were evaluated. The results showed that the inhibitory effect on algal growth (EC_50_-96h) of triclosan varied with 50 times differences among the 7 algal species. *Chlorella ellipsoidea* was the least susceptible species and *Dunaliella parva* was the most sensitive species to triclosan. The inhibitory effect of fluoxetine was less variable than triclosan. Slightly higher toxicity of fluoxetine than triclosan was shown in the 7 tested algal species. No consistent pattern of the effects from mixture of triclosan and fluoxetine was observed among the 7 algal species and among the 4 genera. Additive effects of the mixture occured in 4 species and antagonistic effects in the other 3 species but no synergistic effect was detected. The algal species might show some sign of phylogenetic response to triclosan, as evidenced by the wide range of differences in their sensitivity at the genus level. This study provides important data which could be beneficial for biomonitoring programs on the ecological risk (algal species diversity) of these two chemicals.

## Introduction

Pharmaceuticals and personal care products (PPCPs) are a large group of chemicals including antibiotics, hormones, anti-inflammatory drugs, disinfectants, insect repellants, and UV-filters, etc.^1,2^. Considered as emerging contaminants, PPCPs have been receiving increasing attention in recent years for their occurrence in waters and effects in aquatic organisms^3–5^. In 2011, the global annual production of PPCPs has been estimated at approximately 13 million tons^1^. Due to incomplete removal in wastewater treatment plants (WWTPs), some PPCPs in domestic, industrial and hospital sewages after WWTPs treatment will have the chance to enter the receiving aquatic environments. PPCPs and their metabolites have been detected in various waters at levels that are generally in the ng/L range, sometimes can be found in μg/L level, and some of the PPCPs may be accumulative in aquatic organisms^3,6–8^.

Triclosan (5-chloro-2-(2,4-dichlorophenoxy)phenol) is a synthetic antimicrobial chemical and applied in a variety of consumer healthcare products, soaps and plastics^9^. It has been used as a disinfectant for several decades^10^. It is one of the most frequently detected PPCPs in surface water worldwide^8^. The concentration of triclosan was found in the range of 0.011 to 2.7 μg/L in surface water^8,11^, and the mean concentration was approximately 10 μg/L in untreated wastewaters^12^. After treatment in WWTPs, triclosan concentrations in typical effluents average 0.78 μg/L^13^, which may cause adverse effects in many aquatic organisms^14,15^. Triclosan has a log*K*_*ow*_ value of is 4.8 at pH 7.5 and is likely to be photodegraded in water. However, its degradation products (e.g. 2,4-DCP and 2,8-DCDD) are persistent and more toxic than triclosan itself^16,17^. The hydrophobic property of triclosan increases its potential for bioaccumulation and trophic transfer through food web^18^. The effects of triclosan have been studied in a variety of aquatic organisms^19^. The EC_50_-96h values range from 0.53 to 800.0 µg/L for algae and LC_50_-96h values range from 184.7 to 3000 µg/L for aquatic invertebrates^19–21^. Triclosan blocks the lipid synthesis by inhibiting the enzyme enoyl-acyl carrier protein reductase (ENR)^22,23^ and destabilizing the cell membrane^24^. This may increase disturbance of the permeability-barrier functions on the membrane^25^.

Fluoxetine (N-methyl-3-phenyl-3-[4-(trifluoromethyl)phenoxy]propan-1-amine) is a selective serotonin reuptake inhibitor (SSRI), among the most often prescribed drugs for the treatment of depression and some compulsive disorders for more than two decades^26^. It has been found that fluoxetine concentration was up to 0.012 μg/L in streams in the U.S and concentration was from 0.013 μg/L to 0.099 μg/L in WWTPs effluent^27^. Fluoxetine can be rapidly metabolized to norfluoxetine^28^. Fluoxetine has a log*K*_*ow*_ value of approximately 4.5 in surface water and is hydrolytically and photolytically stable in water. It can be rapidly dissipated from water phase as a result of adsorption in the particulate matter or sediment in natural water^29^. It is persistent in aquatic environments^29,30^. Due to its enantiospecific effects, it may cause potential deleterious effects to aquatic organisms at even low concentrations^31^. EC_50_-96h values of fluoxetine range from 16.0 to 900 µg/L in freshwater algae and LC_50_-96h values range from 234 to 820 µg/L in aquatic invertebrates^32–34^. In addition, fluoxetine can be transferred from the lower trophic level to the higher trophic level in a laboratory-demonstrated three-level aquatic food chain^35^. Exposure to fluoxetine in algae results in cell deformities and smaller sizes at concentrations over 13.6 µg/L^36^. In addition, fluoxetine has also been found to inhibit efflux pumps in cell membrane^37^.

Algae form the base of aquatic food webs and play important roles in energy and nutrient transfer to upper trophic level species. In addition, algae have proved to accumulate many pollutants from the water which can be transferred to species at higher trophic levels^38–40^. Algae have a fast reproduction rate and high sensitivities to environmental disturbance and pollution. Some algal species can be either used as environmental indicator or capable of removing pollutants. For example, species of *Chlorella* and *Scenedesmus* are relatively tolerant to environmental contaminants and are highly efficient in treatment of industrial and household wastewater^41,42^. *Chlamydomonas sp*. is effective for phosphate removal^43^. *Dunaliella* are salt tolerant species found in salt lake and marine environment and are often used as model test species for marine and estuary environments^44,45^.

No single species in general can be expected to represent all other species from the same biological classification unit (i.e., at the order or genus levels) in the response to environmental stressors. Different algae species may have different sensitivity to environmental pollutants. Studies of the PPCPs toxicity to different algae among different genera may increase our understanding of triclosan and fluoxetine toxicity to freshwater algae. Former studies on the effects of triclosan and fluoxetine have focused on their individual effects in a few algal species^19,32,36,46^. The effects of a mixture of these two common chemicals to different algal species have not been studied. However, in the field, these two chemicals are most often detected concurrently in waters receiving effluents from WWTPs^47–49^. In this study, the individual and mixture effects of these two chemicals were determined for 7 algal species from four different genera by growth inhibition bioassays. The objectives of this study were to determine the sensitivity of different algal species to triclosan and fluoxetine, and to determine the joint actions of these two chemicals to the different algal species. The results from this study will broaden our knowledge on the general toxicity of these two chemicals to algae and improve our understanding of the different sensitivities in different algal species among different genera. The outcome of this work will help us understand the ecological risk of the two chemicals.

## Material and Methods

### Chemicals

Triclosan (purity >97%) and fluoxetine (purity >98%) were purchased from Aladdin Industrial Corporation (Shanghai, China) and Sigma-Aldrich (St., Louis, MO, USA) respectively. Dimethyl sulfoxide (DMSO) was purchased from Sigma-Aldrich. All glassware and other containers were acid washed, rinsed with deionized water, air-dried, and autoclaved before use.

### Culturing of Algae

Algal strains in this study were purchased from the Freshwater Algae Culture Collection (FACHB-collection) at the Institute of Hydrobiology, Chinese Academy of Sciences. Seven species were studied from *Chlorella* (*C. pyrenoidosa* and *C. ellipsoidea*), *Scenedesmus* (*S. obliquus* and *S. quadricauda*), *Dunaliella* (*D. salina* and *D. parva*) and *Chlamydomonas* (*C. microsphaera*) genera. The algae were cultured in 250 ml flasks with approximately 100 mL of medium prepared according to the OECD guideline for 5 of the species (*C. pyrenoidosa*, *C. ellipsoidea*, *S. obliquus*, *S. quadricauda*, *C. microsphaera*) (OECD 201) (see Table S1. The recipe of the culture medium from OECD 201 for the recipe of the medium), and in flasks with a medium according to FACHB for two of the species (*D. salina* and *D. parva*) (see Table S2. The recipe of the *Dunaliella* medium. for the recipe of the medium). The cultures of all algal strains were maintained in the lab by re-inoculating in freshly sterilized flasks with freshly prepared medium at 1:20 (v/v) at least once a week. The purity of the algae stock was frequently examined under a microscope connected to a computer with software aiding in algal species counting and identification (Shineso Algacount-Sx, Hangzhou, China).

### Exposures to triclosan and fluoxetine

Preliminary experiments were conducted to determine the appropriate range of exposure concentrations for the determination of EC_50_ for each algal species. The growth inhibition tests for each algal species were performed in 96-well microplates according to the method by Petersen, *et al*.^34^ with some modifications. The nominal exposure concentration range was 0–2000 µg/L for triclosan and 0–1280 µg/L for fluoxetine with 9 concentration gradients including solvent control (i.e., DMSO at approximately 0.1% (v/v) of the exposure volume), respectively. Prior to the exposure, algae cultures were incubated in the growth medium for 4 to 6 days to ensure the cultures to be at the stage of exponential growth with a cell density reaching approximately 10^5^–10^6^ cells /mL. The relationships between absorbance (optical density at 450 nm on a Multiskan FC spectrophotometer, Thermo Scientific, China) and algal concentration (cell density) were assessed for all strains (in all cases, the straight line had a R^2^ > 0.90). In addition, algal density in the stock and the diluted solutions was estimated using a hemocytometer and counted with the aid of the computer software for algal counting (Shineso Algacount-Sx, Hangzhou, China) to ensure the comparability between absorbance and cell density. Results from both methods were comparable.

For all exposures (including individual and mixture), the outer wells of a microplate were filled with 200 µL of growth medium to counteract the edge-specific evaporation from the microplate (Thermo, China)^34^. For each algal culture, the cell concentration was adjusted to 5 × 10^4^ cells/mL from the algal stock in a 10 mL centrifuge tube. For each culture, ten 10 mL tubes were prepared, each containing the diluted algal solution for a treatment in the exposure (see below). Freshly prepared stock solutions of the chemical (triclosan or fluoxetine) were transferred to each culture tube to achieve the nominal exposure concentrations. After that, approximately 200 μL from each tube was transferred directly to a well in a microplate. The exposure for each species was conducted using at least four plates (therefore four replicates). Each plate had 10 exposure concentrations including a control (growth medium only), a solvent control, and 8 concentrations of the chemical (i.e, C_Fi_ for fluoxetine and C_Ti_ for triclosan, where i = 1 to 8, representing 8 concentrations used in the exposure). The wells from the second to the sixth row on the plates were used for the exposure (therefore, on each plate, there were five replicates for each concentration). The wells of the seventh row on the plate contained growth medium and exposed chemical without algal cells. This row was used to monitor the change of absorbance due to the chemical alone during the 96 h exposure (for more direct visualization of the arrangement of the wells in each plate, please see Fig. S1). Each plate was sealed with a plate cover and then wrapped with parafilm before incubated on a microplate shaker (Leopard, China) at 200 rpm. A continuous illuminance was maintained at 1700 ± 100 lux and temperature was maintained at 20 ± 2 °C. After 96 h of exposure, the absorbance of the algal culture was measured at 450 nm on a Multiskan FC spectrophotometer.

For the binary mixture exposure, similar exposure regime was employed except for the chemicals used. Each of the 8 exposure concentrations for the mixture was a simple addition of the concentration of each chemical in its individual exposure (i.e., C_Mi_ = C_Fi_ + C_Ti_, where, i = 1 to 8, representing 8 concentrations used in the exposure; C_Mi_ is the ith concentration used in the mixture, Fi is the individual concentration of fluoxetine, Ti is the concentration of triclosan) (for more direct visualization of the arrangement of the wells in the plate for mixture exposure, please see Fig. S2). After 96 h of exposure, the absorbance of the algal culture was monitored at 450 nm.

### Statistical analyses

All data were expressed as mean ± standard error unless otherwise stated. The concentration-response curve was set from the experimental data for each alga and each chemical exposure (single substance or binary mixture). For the inhibitory effects of individual chemicals, the relative algal growth in each well was fitted to the exponential growth function, using the Graphpad Prism software (Version 5, San Diego, CA, USA). Since there was no intra-plate effect, the measured absorbance values of the five replicated wells in each microplate were combined to generate an arithmetic mean, which was used as a single datum point. Since four plates were used for each algal species, the number of replicates was four. The differences in absorbance for each concentration among the four replicated plates were determined using One-way analysis of variance (ANOVA). Genus, treatment, and species parameters were used as fixed effects to test if the inhibitory effects were significantly different for the fixed factors. *P* < 0.05 was considered to be significantly different.

To determine whether the effect of the binary mixture is additive, antagonistic or synergistic, the statistical method of Marking and Dawson^50^ was adopted using the Eq. (1):

1 $${\rm{S}}=\frac{{{\rm{A}}}_{m}}{{{\rm{A}}}_{i}}+\frac{{{\rm{B}}}_{m}}{{{\rm{B}}}_{i}}$$

where A and B are the two chemicals, i and m are the toxicities (EC_50_’s) of the individual chemicals and the mixture, respectively, and S is the sum of the toxicities. S = 1.0 (within the 95% confidential interval) denotes additive effect. S < 1.0 indicates synergistic effect. S > 1.0 indicates antagonistic effects.

### Ethical approval

This article does not contain any studies with animals performed by any of the authors.

## Results

### Inhibitory effects of individual chemicals in seven algal cultures

**Figure 1:**
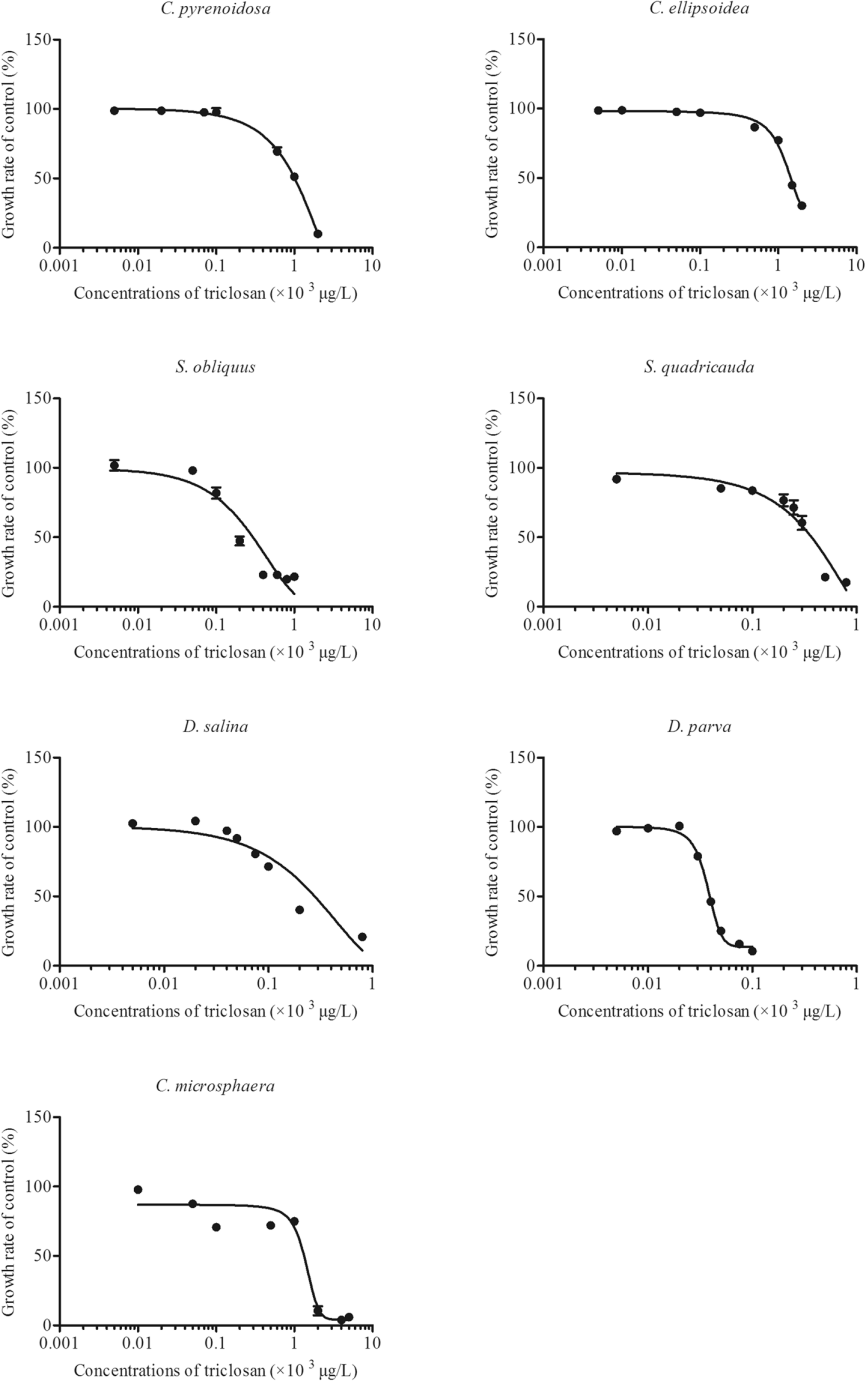
Concentration-response curves of 7 algal species to triclosan. The growth rate of 7 algal species exposed to concentration gradients of triclosan for 96 h relative to that of the control (mean ± standard error, n = 4).

**Figure 2:**
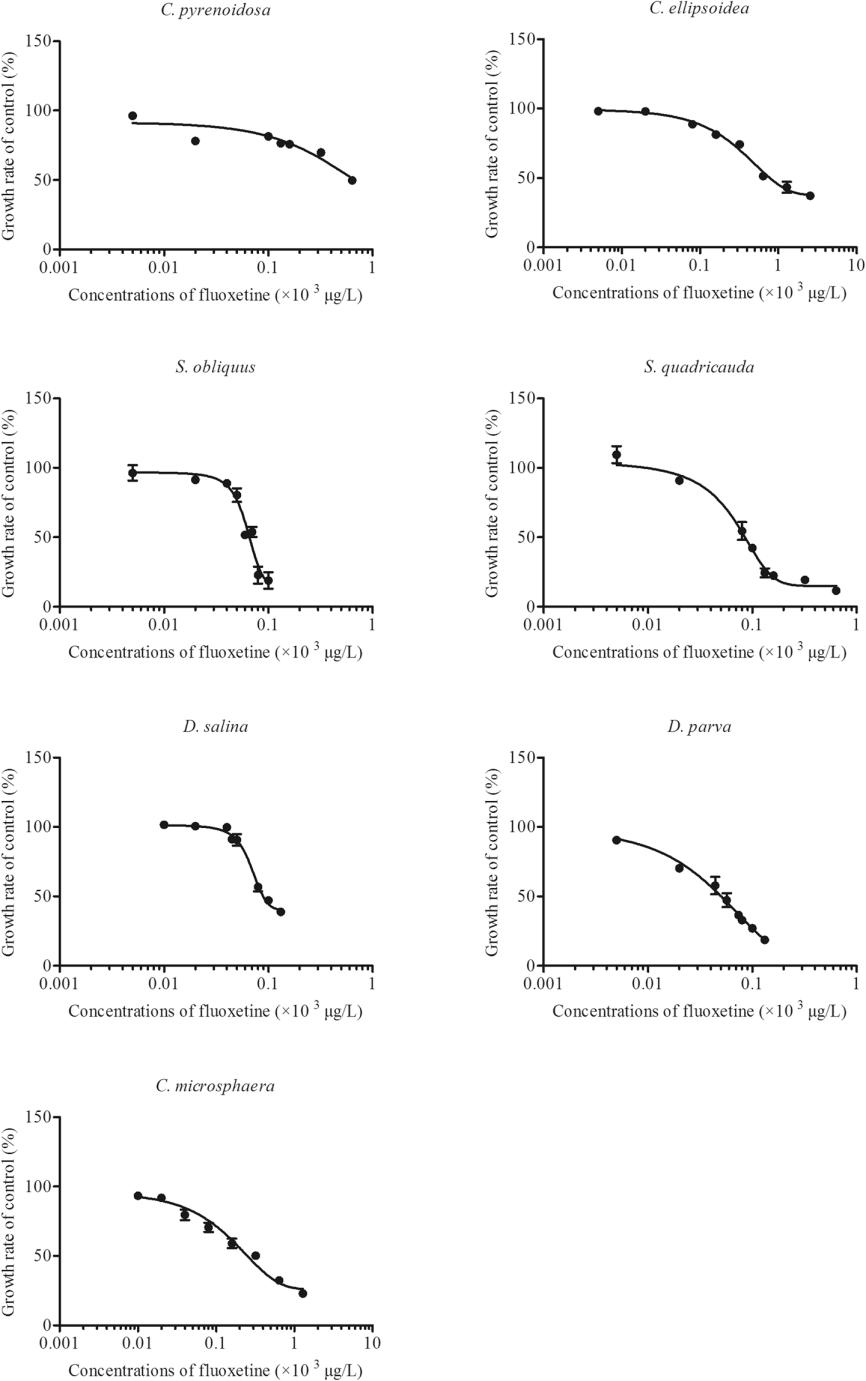
Concentration-response curves of 7 algal species to fluoxetine. The growth rate of 7 algal species exposed to concentration gradients of fluoxetine for 96 h relative to that of the control (mean ± standard error, n = 4).

**Figure 3:**
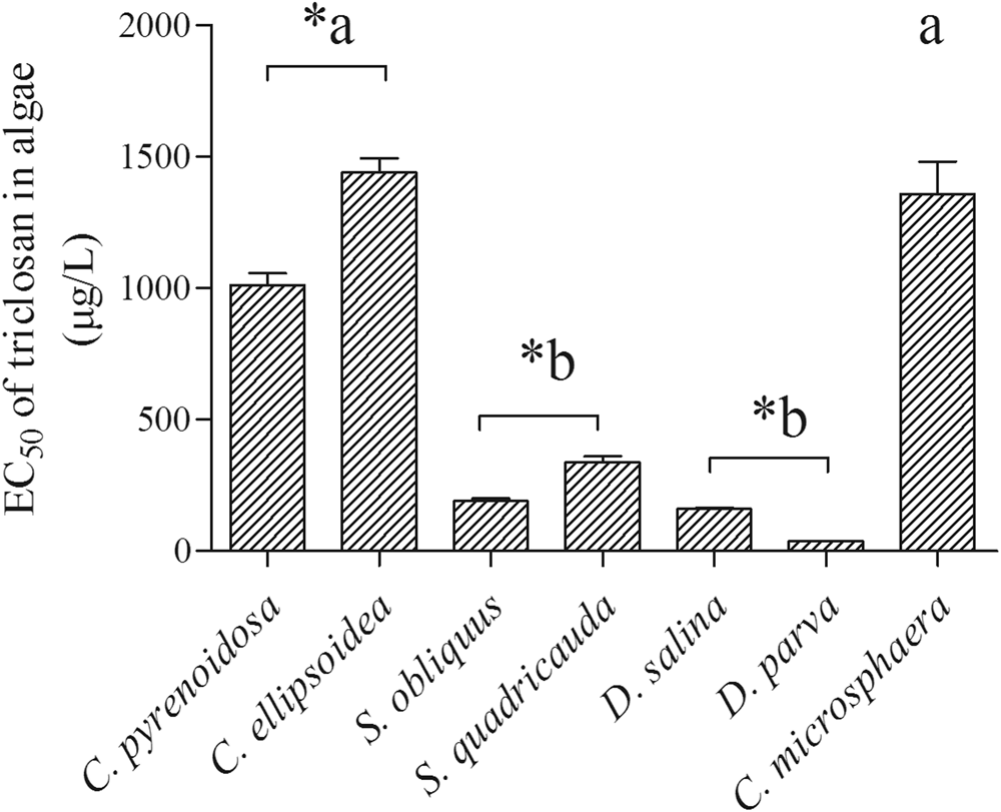
The EC50 of 7 algal species exposed to triclosan for 96 h. And mean ± standard error, n = 4. Asterisk (*) indicated significant difference (*P* < 0.05) within the same genus. The letters indicated significant differences (*P* < 0.05) among genera.

In general, the concentration response (CRC) data on the relative growth of each algal strain were fitted well to the non-linear regression line (R^2^ > 0.90, *p* < 0.001 in all cases) for both chemicals (Figs 1 and 2). For triclosan, the no-observed effect concentrations (NOEC) in the seven algal species ranged from 6.2 µg/L in *S. quadricauda* to 100 µg/L in *C. pyrenoidosa* and *C. ellipsoidea* (Table 1). The lowest observed effect concentrations (LOEC) spanned from 9 µg/L in *C. microsphaera* and 600 µg/L in *C. pyrenoidosa* (Table 1). There was a significant difference in the susceptibility to triclosan among the four genera (ANOVA, F_(3,12)_ = 23.8, *p < *0.0001). The susceptibility to triclosan for the four genera ranked as *Dunaliella* ~ *Scenedesmus* > *Chlorella* ~ *Chlamydomonas*. The EC_50_-96h varied with a factor of approximately 50 times among the seven algae species (Fig. 3). *C. ellipsoidea* was the least susceptible species with an EC_50_-96h of 1441.5 ± 52.8 µg/L, while *D. parva* was the most sensitive species with an EC_50_-96h of 39 ± 0.1 µg/L (Fig. 3). Algal species in the same genera showed different susceptibilities to triclosan in 96 h exposure. *C. pyrenoidosa, S. quadricauda, D. salina* were more susceptible than *C. ellipsoidea, S. obliquus and D. parva*, respectively (Fig. 3).

**Table 1:** The 96 h NOEC and LOEC values based on growth inhibition of 7 algal species exposed to a concentration gradient of triclosan or fluoxetine.

Genera	Species	Triclosan (µg/L)		Fluoxetine (µg/L)	
		NOEC	LOEC	NOEC	LOEC
*Chlorella*	*C. pyrenoidosa*	100	600	6.2	18.6
	*C. ellipsoidea*	100	500	18.6	80.4
*Scenedesmus*	*S. obliquus*	49	100	40.2	49.5
	*S. quadricauda*	6	49	18.6	80.4
*Dunaliella*	*D. salina*	41	49	40.2	46.4
	*D. parva*	20	29	6.2	18.6
*Chlamydomonas*	*C. microsphaera*	n.d.	9	18.6	40.2

n.d.: data was not available.

**Figure 4:**
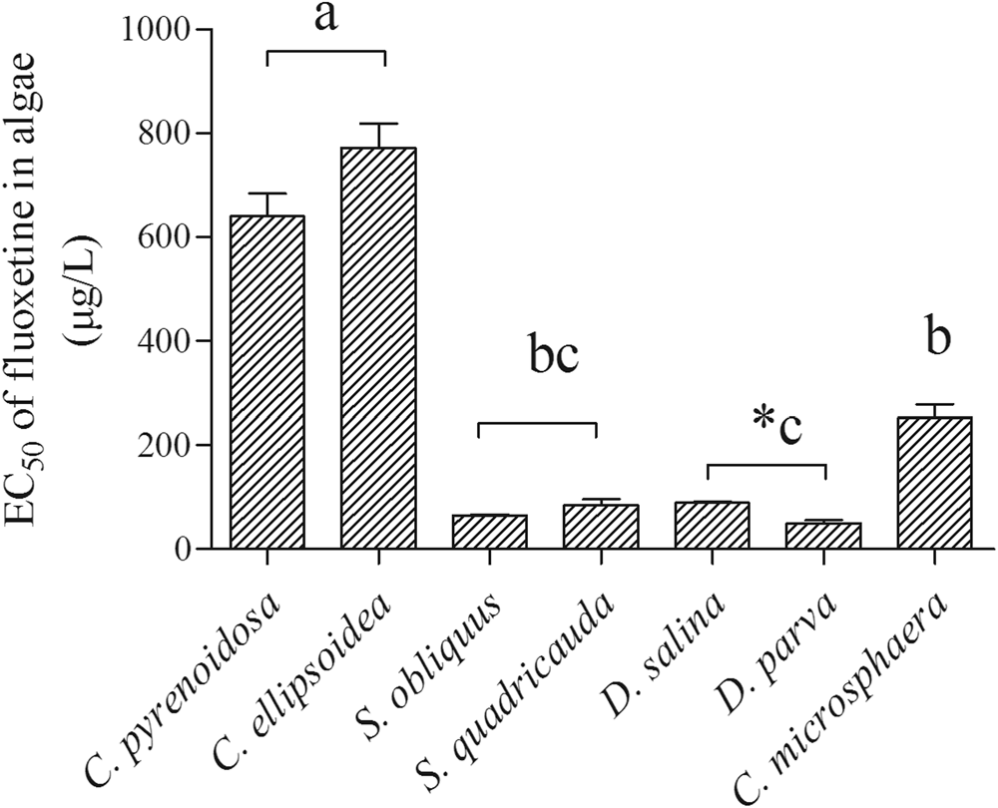
The EC50 of 7 algal species exposed to fluoxetine for 96 h. And mean ± standard error, n = 4. Asterisk (*) indicated significant difference (*P* < 0.05) within the same genus by Turkey’s test. The letters indicated significant difference (*P* < 0.05) among genera.

For fluoxetine, the NOEC in the seven algal species varied from 6.2 µg/L in *C. pyrenoidosa* and *D. parva* to 40.2 µg/L in *S. obliquus* and *D. salina* (Table 1). The LOEC had a range from 18.6 µg/L in *C. pyrenoidosa* and *D. parva* to 80.4 µg/L in *C. ellipsoidea* and *S. quadricauda* (Table 1). There was a significant difference among different genera in the susceptibility to fluoxetine (ANOVA, F_(3,12)_ = 64.1, *p* < 0.0001), with *Chlorella* being the least sensitive genus, followed by *Chlamydomonas*. The EC_50_-96h values were less variable than those of triclosan, with approximately 13 times of differences among the seven algal species (Fig. 4). *C. ellipsoidea* and *C. pyrenoidosa* were the two least susceptible species with EC_50_-96h values of 640.0–773.3 µg/L, followed by *C. microsphaera*. The other four species had similar susceptibility to fluoxetine. There was no significant difference in algae responses in fluoxetine within the same genera, except that *D. parva* was more susceptible than *D. salina* to fluoxetine in 96 h exposure time (Fig. 4).

Finally, the EC_50_-96h values of fluoxetine were generally lower than those of triclosan in the 7 algal species. At the species level, fluoxetine showed higher inhibitory effects than triclosan (*p* < 0.001) in six of the seven tested species, except for *D. parva*, which had similar EC_50_ values for the two chemicals (Figs 3 and 4).

### Effects of binary mixture of triclosan and fluoxetine in seven algal cultures

The sum of toxicity (S) of the mixture of triclosan and fluoxetine in the 7 algal species ranged from 0.9–2.0 (Table 2). In general, no consistent pattern of the effects of the binary mixture was observed among the 7 algal species and among the 4 genera studied. Both antagonistic and additive effects were noticed for the binary mixture in the 7 species. Additive effects of the mixture ocurred in 4 species and antagonism in the other 3 species (Table 2). However, no synergistic effect was observed in all the seven algal species.

**Table 2:** Sum of toxicity (S) of the mixture of triclosan and fluoxetine in the 7 algal species.

Genera	Species	S (mean and 95% CI)	Interactions
*Chlorella*	*C. pyrenoidosa*	1.3 (0.96–1.66)	antagonistic
	*C. ellipsoidea*	1.0 (0.73–1.20)	additive
*Scenedesmus*	*S. obliquus*	2.0 (1.64–2.42)	antagonistic
	*S. quadricauda*	1.1 (0.46–1.73)	additive
*Dunaliella*	*D. salina*	1.5 (1.26–1.73)	antagonistic
	*D. parva*	0.9 (0.57–1.24)	additive
*Chlamydomonas*	*C. microsphaera*	1.2 (0.58–1.78)	additive

The effect of the two chemicals in mixture is supposed to be additive when S is not different from 1.0; synergistic when S is less than 1.0; and antagonistic when S is greater than 1.0 (n = 4).

## Discussion

In this study, both triclosan and fluoxetine showed inhibitory effects in the seven algal species within 96 h of exposure. The inhibitory effects of triclosan had a relatively wide range (50 times of differences) among the seven species. The inhibitory effects for triclosan in 5 of the 7 algal species (except for *C. Pyrenoidosa* and *S. obliquus*) have never been reported in the literature. The EC_50_-96h values for triclosan in algae have a relatively wide range from 0.53 (in *Pseudokirch-neriella subcapitata*) to 800 µg/L (in *C. pyrenoidosa*) in algae^15,20,21,51^. Metabolic pathway of triclosan may include biodegradation, hydroxylation, methylation, gluocosylation and xylosylation in different organisms, where the major pathyway for triclosan is biodegradation in *C. pyrenoidosa*, and biotransformation in *S.obliquus*^51,52^. Other factors, such as algal species, culture medium, pH, light intensity would also affect the inhibitory effect of triclosan on the growth of algae^15,19,36,53,54^. Since triclosan targets lipd synthesis^23^, the algal susceptability to triclosan will be highly depending on the interaction of triclosan to the plant ENR and the binding of FabI proteins to triclosan^55,56^. Differences in algal physiology and metabolic pathway of triclosan could contriubute to the difference in toxicity. The concentration of triclosan was observed to decrease rapidly by 50% within 1 h after *C. pyrenoidosa* was incubated in its culture medium containing 800 µg/L of triclosan^21^. The formation of coenobia of *Scenedesmu* is possible under environmental stresses, and large colonies would eventually reduce surface-to-volume ratio and limit nutrient uptake and light harvesting, thus inhibit the growth rate^57^. Both *D. salina* and *D. parva* were more susceptible to triclosan than other tested species in the present study, whose EC_50_-96h values was 162 and 41 μg/L, respectively. The EC_50_-96h of triclosan toxictity to another marine alga *D.tertiolecta* was 3.5 μg/L^44^. The activity of internally duplicated carbonic anhydrase (Dca) in *D. salina* stress protein (60-kDa) was higher than that found in *D. tertiolecta* and *D. parva*^58,59^, which helps to explain the higher tolerance in *D. salina* than *D. parva* and *D. tertiolecta*.

The above mentioned factors can help partially explain the relatively wide range of EC_50_-96h values for triclosan in algae reported in the literature. However, the EC_50_-96h values in *C. pyrenoidosa* (1000 μg/L) in this study was comparable with that (~810 μg/L) of the same species in a previous study^21^. The comparison of the EC_50_-96h values for the other tested species in the current study with the data from literatures was not possible, due to the paucity of data on the sensitivity to triclosan from other algal species. This reinforces the necessity of future studies on the effects of triclosan in more algal species.

The inhibitory effects of fluoxetine were less variable than triclosan, only 13 times of differences among the seven species were found. Unlike triclosan, fluoxetine concentrations in the exposure media was relatively stable in the growth medium during the 96 h exposure time^26^. The EC_50_-96h values of the seven algae in this study were comparable with those from previous studies^32,60^. For example, the EC_50_-96h value of the same species *S. quadricauda* was estimated to be 177 ± 13.3 μg/L using cell density as the endpoint^61^, which was approximately 2 fold of its EC_50_-96h (~93 μg/L) in this study.

In *D. tertiolecta*, triclosan was more toxic than fluoxetine^44^. However, in the present study, fluoxetine was slightly more toxic to algae than triclosan. The possible reasons could be that: 1) triclosan was biodegraded or biotransformed, which reduced its toxicity^21,51^; 2) fluoxetine was metabolized to norfluoxetine, which is more toxic that the parent compound^28^. This is also in agreement with the previous studies that the EC_50_-96h value of fluoxetine and triclosan in *Skeletonema pseudocostatum* was approximately 15.0 μg/L and 30 μg/L, respectively^34,62^. Moreover, the tested agal species showed inconsistent sensitivities to triclosan among species and genera. Triclosan may have multiple target sites or undergo various degradation and transformation in different algal species compared to fluoxetine. Although the mechanisms of triclosan and fluoxetine toxicities to individual algal species remain unknown, the differences in chemical properties and algae sensitivities might give us some hints for understanding the different response in algal species among the tested genera.

It is likely that fatty acid metabolism in plants might also be disrupted by the same mechanism due to the similarity in fatty acid synthesis between bacteria and plants^62^. For fluoxetine, its toxicity to algae might be related to its ability to disrupt membrane protein binding processes in a nonspecific way^30^. In addition, it is possible that fluoxetine exerts toxicity in organisms by inhibiting cell efflux pumps^37,44^. To certain extent, triclosan and fluoxetine may both have disrupting effect on the cell membrane. Further research is needed to better elucidate the mechanistic pathway of the two chemicals to different algal species.

Chemicals rarely exist individually in the nature, the study of combined effects of chemicals might help us better understand the toxicities of mixture. Our results showed that additive effects of the binary mixture in four of the seven algal species and antagonistic effects were found in 3 species. Similar to our results, the mixture of triclosan and fluoxetine showed additive effects in *D. tertiolecta*^44^. The antagonistic/synergistic/additive effects of mixture can be species specific. For example, in a study on the effects of eight mixtures of herbicides (with different sites of action) in six plants and algal species, it was shown that only two of the mixtures were consistently antagonistic across all species studied, while for the remaining six mixtures, the joint effect depended on the species tested^63^.

Finally, the susceptibility of the seven algae to triclosan showed a relatively wide range (but less to fluoxetine) and both chemicals showed significant differences in susceptibility at the genus level but similar at species level. Previous studies have shown that sensitivity to chemical pollutants (including organic pollutants and metals) might show phylogenetic signals in aquatic organisms^64–66^. For example, significant phylogenetic signal was demonstrated in the sensitivity to four herbecides (atrazine, terbutryn, diuron, and isoproturon) among 14 diatom species representative of a freshwater lake^64^. Phylogenetic signal on the susceptiblity to pollutants can help explain the field observations in the contaminated environments. For example, Buchwalter *et al*. showed that susceptiblity to Cd among 21 aquatic insects showed a significant phylogenetic signal in a laboratory study^65^. The absence and presence of aquatic insects along the metal gradients in the mining impacted Clark River, USA^67^, corresponded to the sensitivities of these species to Cd in their respective clades assessed in the laboratory, with the most Cd sensitive mayfly species that dissappeared earlier than the less susceptible caddisfly species. Due to the requirement of large database to detect the phylogenetic signal in sensitivity to chemical pollutants in organisms, it could not be concluded from this study whether the sensitivity to these two chemicals had a phylogenetic basis. Meanwhile, the use of a phylogenetic framework to improve biomonitoring remains an unexplored field, and few data are available for freshwater algae^64,68^. Based on the wide range of differences in sensitivity of the 7 algal species to the two chemicals (especially for triclosan) in this study, it will be very interesting to include more species in future work to test whether a phylogenetic signal exists for the susceptibility to these two chemicals in algae. It is suggested that approaches that integrate phylogeny and ecotoxicology can provide information for bioassessment tools operating at a larger taxonomic scale and thus increase the effectiveness of biomonitoring^68^. Up to now, the lack of quality datasets based on multiple species with a wide range of sensitivities has hindered this type of research^69^. Such quality datasets may also bring valuable sensitivity data to build relevant risk assessment models such as Species Sensitivity Distribution^70^ in order to predict efficiently effects of PPCPs and their mixtures.

This study deomonstrated that both fluoxetine and triclosan can inhibit the growth of seven algae. Fluoxetine seemed to have higher inhibitory effects on the growth of the algae than triclosan. The binary mixture showed additive and antagonistic effects depending on the algal species. Future research could focus on the mechanisms of effects (inhibition of growth and other detrimental effects) of the two chemicals in algae and on the determination of the sensitivity to these two chemcials using more species from different genera in order to understand the response of agal species to the chemical eposure and consequent change in structure and function of phytoplankton community, in order to predict the risk of PPCPs to the aquatic ecosystems.

## Electronic supplementary material

Supplementary Information

## Data Availability

The datasets analyzed during the current study are available from the corresponding author on reasonable request.
