# Supplementary material for: Sensitivities of seven algal species to triclosan, fluoxetine and their mixtures
Source: Sci Rep. 2018 Oct 18;8:15361. doi: 10.1038/s41598-018-33785-1 (PMC6193942; doi:10.1038/s41598-018-33785-1)
Supplement: Supplementary file 1 — Supplementary Information [file 41598_2018_33785_MOESM1_ESM.pdf]

Sensitivities of seven algal species to triclosan, fluoxetine and their mixtures

Ran Bi[1*], Xiangfeng Zeng[2*], Lei Mu[2], Liping Hou[3], Wenhua Liu[1], Ping Li[1], Hongxing Chen[4], Dan Li[2], Agnes Bouchez[5], Jiaxi Tang[6] and Lingtian Xie[4]

1. Marine Biology Institute, Shantou University, Shantou, Guangdong 515063, China
2. Key Laboratory of Pollution Ecology and Environmental Engineering, Institute of Applied Ecology, Chinese Academy of Sciences, Shenyang, Liaoning 110016, China
3. School of Life Sciences, Guangzhou University, Guangzhou, Guangdong 510655, China
4. The Environmental Research Institute, MOE Key Laboratory of Environmental Theoretical Chemistry, South China Normal University, Guangzhou 510006, China
5. Institute National de la Recherche Agronomique, UMR CARRTEL, Thonon-les-Bains 74200, France
6. College of Environmental Science and Engineering, Liaoning Technical University, Fuxin, Liaoning 123000, China

Table S1 The recipe of the culture medium from OECD201

	Component	Dosage
1	NH_4Cl	15 mg/L
2	$MgCl_2 \cdot 6H_2O$	12 mg/L
3	$CaCl_2 \cdot 2H_2O$	18 mg/L
4	$MgSO_4 \cdot 7H_2O$	15 mg/L
5	KH_2PO_4	1.6 mg/L
6	$FeCl_3 \cdot 6H_2O$	0.08 mg/L
7	$Na_2EDTA \cdot 2H_2O$	0.1 mg/L
8	H_3BO_3	0.185 mg/L
9	$MnCl_2 \cdot 4H_2O$	0.415 mg/L
10	$ZnCl_2$	3×10^{-3} mg/L
11	$CoCl_2 \cdot 6H_2O$	1.5×10^{-3} mg/L
12	$CuCl_2 \cdot 2H_2O$	10^{-5} mg/L
13	$Na_2MoO_4 \cdot 2H_2O$	7×10^{-3} mg/L
14	$NaHCO_3$	50 mg/L

Table S2. The recipe of the *Dunaliella* medium.

	Component	Dosage
1	NaCl	87.69 g/L
2	$NaNO_3$	0.42 g/L
3	$NaH_2PO_4·2H_2O$	0.0156 g/L
4	$CaCl_2·2H_2O$	0.044 g/L
5	KCl	0.074 g/L
6	$MgSO_4·7H_2O$	1.23 g/L
7	$NaHCO_3$	0.84 g/L
8	Ferric citract(1%)	0.5 mL
9	A5*（Trace mental solution）	1mL/L

A5(Trace mental solution)

#	Component	Dosage
1	H_3BO_3	2.86 g/L
2	$MnCl_2·4H_2O$	1.86 g/L
3	$ZnSO_4·7H_2O$	0.22 g/L
4	$NaMoO_4·2H_2O$	0.39 g/L
5	$CuSO_4·5H_2O$	0.08 g/L
6	$Co(NO_3)_2·6H_2O$	0.05 g/L

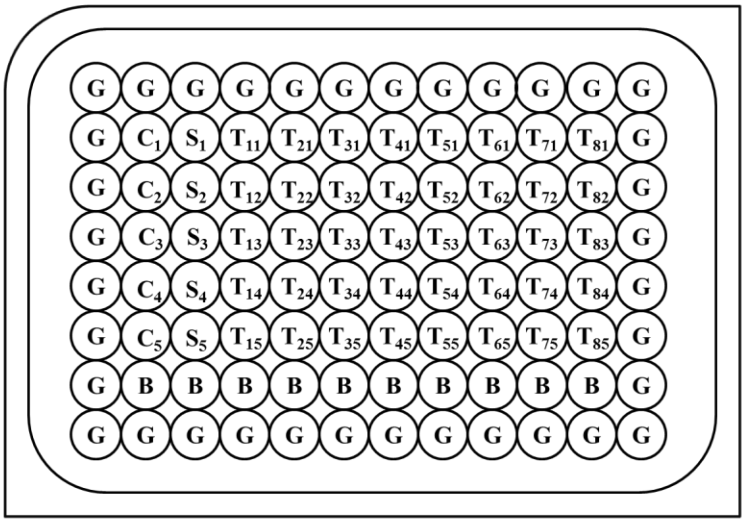

Fig S1. A typical placement for wells of the individual exposure (triclosan or fluoxetine) in a 96-well plate. Wells with the letter “G” are filled with culture medium only. Wells with letter “B” are filled with the mixture of culture medium and the exposure chemical (triclosan or fluoxetine) without algae cells. C_i (i = 1 - 5) denotes the control wells with algae only. S_i (i = 1 - 5) is the solvent control wells with algae and carrier solvent DMSO (approximately 0.1% v/v). T_{ji} (j = 1 - 8; I = 1 - 5) is the wells for all the treatments, in which j (j = 1 - 8) is the number of concentration and i (i = 1 - 5) is number of replicate for each concentration.

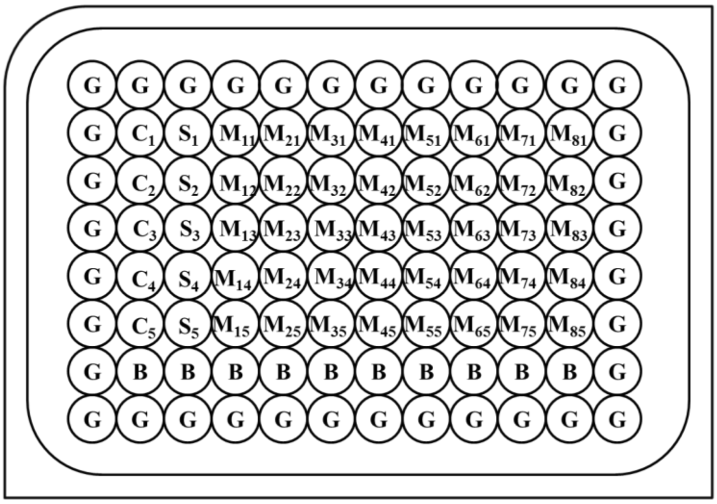

Fig S2. A typical placement for wells of the binary mixture exposure (triclosan + fluoxetine) in a 96-well plate. Wells with the letter “G” are filled with culture medium only. Wells with letter “B” are filled with the mixture of culture medium and the exposure chemical (triclosan + fluoxetine) without algae cells. C_i (i = 1 - 5) denotes the control wells with algae only. S_i (i = 1 - 5) is the solvent control wells with algae and carrier solvent DMSO (approximately 0.1% v/v). M_{ji} (j = 1 - 8; I = 1 - 5) is the wells for all the treatments, in which j (j = 1 - 8) is the number of concentration and i (i = 1 - 5) is number of replicate for each concentration. Each M_{ji} contains the concentration of triclosan and fluoxetine at their respective concentration for their individual exposure (for example, $M_{62} = T_{62} + F_{62}$, T is short for triclosan and F is for fluoxetine).
